# Responsible modelling: Unit testing for infectious disease epidemiology

**DOI:** 10.1016/j.epidem.2020.100425

**Published:** 2020-12

**Authors:** Tim C.D. Lucas, Timothy M Pollington, Emma L Davis, T Déirdre Hollingsworth

**Affiliations:** a Big Data Institute, Li Ka Shing Centre for Health Information and Discovery, University of Oxford, UK. Centre for Environment and Health, School of Public Health, Imperial College, UK; b Big Data Institute, Li Ka Shing Centre for Health Information and Discovery, University of Oxford, UK. MathSys CDT, University of Warwick, UK; c Big Data Institute, Li Ka Shing Centre for Health Information and Discovery, University of Oxford, UK

**Keywords:** Unit testing, Software development, Reproducible science, Computational models

## Abstract

Infectious disease epidemiology is increasingly reliant on large-scale computation and inference. Models have guided health policy for epidemics including COVID-19 and Ebola and endemic diseases including malaria and tuberculosis. Yet a coding bug may bias results, yielding incorrect conclusions and actions causing avoidable harm. We are ethically obliged to make our code as free of error as possible. Unit testing is a coding method to avoid such bugs, but it is rarely used in epidemiology. We demonstrate how unit testing can handle the particular quirks of infectious disease models and aim to increase the uptake of this methodology in our field.

## Highlights

• Unit testing can reduce the number of bugs in code but is rarely used in our field.
• We present a worked example of adding unit tests to a computational model.
• Specific issues such as stochastic code are common in infectious disease modelling.
• Unit testing can handle particular quirks of infectious disease models.
• We hope to increase the use of unit testing in infectious disease epidemiology.

## 1 Introduction

Modelling is an important tool for understanding fundamental biological processes in infectious disease dynamics, evaluating potential intervention efficacy and forecasting disease burden. At the time of writing, infectious disease modellers are playing a central role in the interpretation of available data on the COVID-19 pandemic to inform policy design and evaluation (IHME COVID-19 health service utilization forecasting team and Murray, 2020; Ferguson et al., 2020; Hellewell et al., 2020). Similarly, policy on endemic infectious diseases, such as duration and frequency of control programmes and spatial prioritisation, is also directed by models (Behrend et al., 2020). Such research builds on a long history of modelling for policy (Heesterbeek et al., 2015) and a general understanding of the dynamics of infectious disease systems.

Given the importance of modelling results, it is vital that the code they rely on is both coded correctly and trusted. Bugs can be caused by typos, code behaving in unexpected ways, or logical flaws in the construction of the code. Outside of epidemiology, bugs have been found in code that had been used by many researchers (Neupane et al., 2019) and may lead to retractions (American Society of Clinical Oncology, 2016). Bugs have also been found in highly influential work; a paper that informed austerity policies globally was found to have a crucial computational mistake (Herndon et al., 2014). In engineering, bugs caused the Mars Climate Orbiter and the Mariner 1 spacecraft to become lost or destroyed (NASA, 2020; Board, 1999). We do not know of high profile cases of infectious disease models being found to have bugs once published, but as code is not always shared and little post-publication testing of code occurs, this likely represents a failure of detection. The issue of trust was highlighted recently when Neil Ferguson, one of the leading modellers informing UK COVID-19 government policy, tweeted:

“I’m conscious that lots of people would like to see and run the pandemic simulation code we are using to model control measures against COVID-19. To explain the background - I wrote the code (thousands of lines of undocumented C) 13+ years ago to model flu pandemics…” (Ferguson, 2020).

The code that was released did not include any tests (Ferguson and MRC Centre for Global Infectious Disease Analysis, 2020) but subsequent work has added documentation, while independent code reviews have supported the results of the study (Eglen, 2020; BCS, The Chartered Institute for IT 2020). The tweet and lack of tests garnered considerable backlash (some of which may have been politically motivated (Chawla, 2020)), with observers from the software industry noting that code should be both documented and tested to ensure its correctness (BCS, The Chartered Institute for IT 2020). It is understandable that during the fast-moving, early stages of a pandemic, other priorities were put above testing and documenting the code. It is also important to note that a lack of tests is not unusual in our field, or for some of the authors of this article. To guard against error, policy-makers now standardly request analyses from multiple modelling groups (as is the case in the UK for COVID-19 (SPI-M, 2020)) as a means to provide scientific robustness (both in terms of model uncertainty and in terms of implementation) (Den Boon et al., 2019; Hollingsworth and Medley, 2017), yet this is not enough if the models themselves lack internal validity.

Infectious disease modellers are rarely trained as professional programmers (BCS, The Chartered Institute for IT 2020) and recently some observers have made the case that this has been due to a lack of funding (Baker, 2020). Epidemiological groups such as RECON (Csardi et al., 2020), and broader groups such as rOpenSci (www.ropensci.org), have however started providing support for scientists to develop better coding practices. The communities built around these groups are an invaluable resource for new programmers. It is also notable that while a number of articles have stated that unit tests should be written (Osborne et al., 2014; Wilson et al., 2014; Csardi et al., 2020) there are few texts available that demonstrate the use of unit testing to check infectious disease models. While the basic premise of unit testing is simple, there is an art to knowing what aspects of code can and should be tested. Guides that enable researchers to acquire this skill quickly will benefit the field.

Whilst there are many drivers and attempts to address the problem with code robustness, today’s models are increasingly moving from mean-field ordinary differential equation approximations to individual-based models with complex, data-driven contact processes (Willem et al., 2017; Ferguson et al., 2006). These increases in model complexity are accompanied by growing codebases. Furthermore, while there are some general packages for epidemiological modelling (Jenness et al., 2018; Santos and Fernando, 2020), it is very common for epidemiologists to study a new model and to therefore code it from scratch. Unlike, established packages that have had time to mature and fix many bugs, newly programmed models are more prone to errors. As the mathematical methods depend increasingly on numerical solutions rather than analytical pen-and-paper methods, it becomes more difficult to tell if a bug is present based on model outputs alone. Furthermore, checking models in an *ad hoc* way is biased as unexpected results trigger careful checks of the code while models that show expected behaviour are more likely to be assumed bug-free.

*Unit testing* is a formally-defined, principled framework that compares outputs from code to what the programmer expected to happen (Chapter 7 of Wickham (2015), Osborne et al. (2014); Wilson et al. (2014)). Ready-to-run frameworks for unit testing are available in *R* (R Core Team, 2018), *Julia* (Bezanson et al., 2017a) and *Python* (Python Core Team, 2015a) and are standard practice in the software industry. These testing concepts also apply to many other scientific fields, but here we focus on infectious diseases. Infectious disease modelling presents specific challenges, such as stochastic outputs (Ševčíková et al., 2006; Guderlei and Mayer, 2007; Patrick et al., 2017), which are difficult to test and not covered in general unit testing literature. There are a number of other programming techniques that should be used in conjunction with unit testing, such as defensive programming, version control, pair-programming and comprehensive documentation (Osborne et al., 2014; Wilson et al., 2014; Wickham, 2019, 2015; Csardi et al., 2020) and these are important complements to the methods in this paper. In this primer we introduce unit testing with a demonstration of an infectious disease model. While we use *R* throughout to exemplify the unit testing framework, the concepts apply equally well to the various languages commonly used by modellers such as *Julia* and *Python*; we therefore briefly direct the reader towards available testing frameworks for those languages in Section 7.

## 2 Unit testing foundations

At the heart of every *unit test* is a function output, its known or expected value and a process to compare the two. For the square root function ($\sqrt{x}$, or sqrt(x) in *R*), we could write a test that runs the function for the number 4, i.e. sqrt(x = 4), and compares it to the correct answer i.e. 2. However, often function arguments will cover an infinite range of possibilities and we cannot exhaustively check them all. Instead we devise tests that cover standard usage as well as *special case* scenarios: what do we want our function to do if given a negative number e.g. sqrt(-1), or a vector argument containing strings or missing values e.g. sqrt(c(4, "melon", NA))?

Strictly-defined, unit testing tests code with no dependencies outside of the test definition. This is in contrast to integration testing that tests how these small units integrate with other units of code, including dependencies. Testing at even higher levels includes system testing (which tests how multiple systems such as software and hardware interact) and acceptance testing (in which end-users, or software commissioners, test that it meets requirements). Within the scientific community however, the term unit testing is typically used in a slightly vague way and implies a combination of integration and (strict) unit testing. As so much scientific software relies on various dependencies, even at very low levels, the strict definition of unit testing is not necessarily useful. Here, we continue to use this vague definition, simply focussing on testing of code at a low level. The first benefit of this is that it allows you to work out the exact expected result of a function call. Second, if you do find bugs, they are easier to isolate and fix if you are working at these low levels. Third, code that either calls the low-level functions or relies on outputs from them is easier to test and debug.

In *R*, the testthat package (Wickham, 2011), provides a simple interface for testing. While a variety of test functions can make different comparisons, the two main ones are expect_true() and expect_equal(). expect_true() takes one argument: an expression that should evaluate to TRUE. For the square root example above, we would write expect_true(sqrt(4) = = 2). expect_equal() takes two arguments, an expression and the expected output; so we would write expect_equal(sqrt(4), 2).

There are a number of ways to incorporate unit testing into your programming workflow.

1 Each time you write code for a new, discrete chunk of functionality, you should write tests that confirm it does what you expect. These tests should be kept with the code it is testing (in the same directory or in a subdirectory).
2 Whenever a bug is found outside of the existing testing framework, a new test should be written to capture it. Then if the bug re-emerges it will hopefully be quickly flagged so that the developer can fix it.
3 All of these tests should be run regularly as you develop new code. If a change causes the previous tests to break, this indicates the introduction of an error in the new code, or implies that the older code could not generalise to the adapted environment.

## 3 An example multi-pathogen re-infection model

**Fig. 1:**
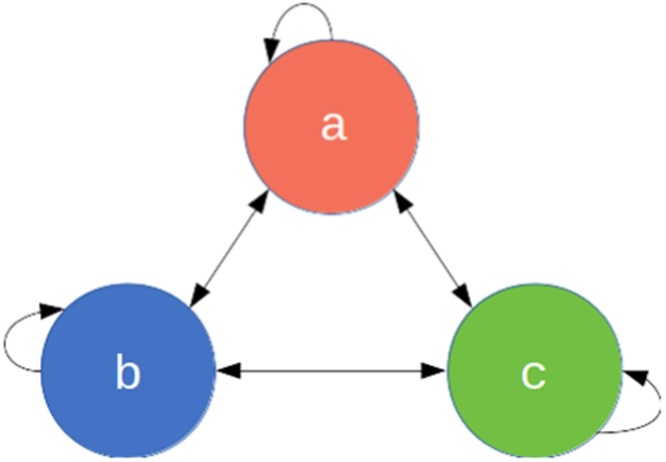
The 3-pathogen system with arrows showing the possible transitions at every time step. A diagram showing 3 compartments, A, B and C. Bidirectional arrows go between each compartment and from each compartment to itself (e.g. A to A).

Here we define a toy epidemiological model and then demonstrate how to effectively write unit tests for it in *R* code. Consider a multi-pathogen system, with a population of N infected individuals whom each get infected by a new pathogen at every time step (Fig. 1). In this toy example, we imagine that individuals are infected with exactly one pathogen at a time. Some aspects of this model could reflect the dynamics of a population where specific antibiotics are used regularly i.e. each time step an individual is infected, diagnosed and treated suboptimally, leaving the individual susceptible to infection from any pathogen, including the one they were just treated for. The aim of this model however is not to be realistic but to serve as a learning tool with succinct code. We work through a more realistic model in the Supplementary Material.

Each individual i, at time t, is defined by the pathogen they are currently infected with $I_{it}\in \{a,b,c\}$ for a 3-pathogen system. The population is therefore defined by a length N state vector $I_{t}={\left(I_{it}\right)}_{i=\left[1,N\right]}$. At each time step, every individual’s infection status is updated as:

$$I_{it}=\text{Unif}\left(I_{t-1}\right).$$

That is, at each iteration, the new infection status of each individual is a Uniform random sample from the set of infection statuses in the previous iteration (including itself $I_{i,t-1}$). This model has a total of three parameters, the total number of individuals, the number of pathogen species, and the number of time steps, all of which are scalar, positive integers. Certainly this toy model is naïve as it is governed by mass-action principles, ignoring contact and spatial dynamics. Nevertheless it will serve its purpose. Before writing any code we can consider the model’s expected behaviour. Firstly, we would expect an individual to be repeatedly infected with different strains. Secondly, we would expect the proportions of the different pathogens to stochastically drift, until all but one pathogen goes extinct. Code 1 shows our first attempt at implementing this model.

Code 1: Base example of the multi-pathogen re-infection model.

**Figure:**
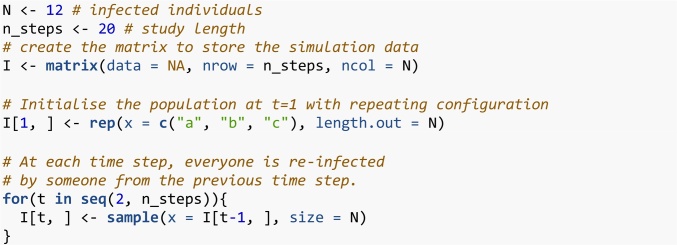

**Fig. 2:**
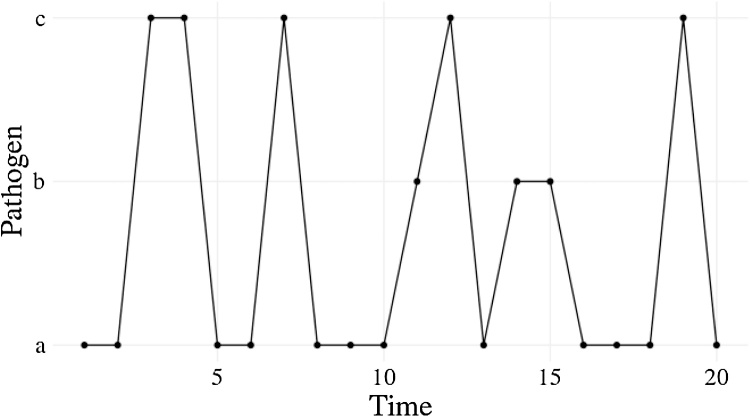
Infection profile for individual 1, who is initially infected with pathogen a but then reinfected with different pathogens. Line plot with time on the x-axis and pathogen (either a, b, or c) on the y-axis. Individual 1 is initially infected with pathogen a, then becomes infected with pathogen c, then continues to be reinfected with different pathogens.

**Fig. 3:**
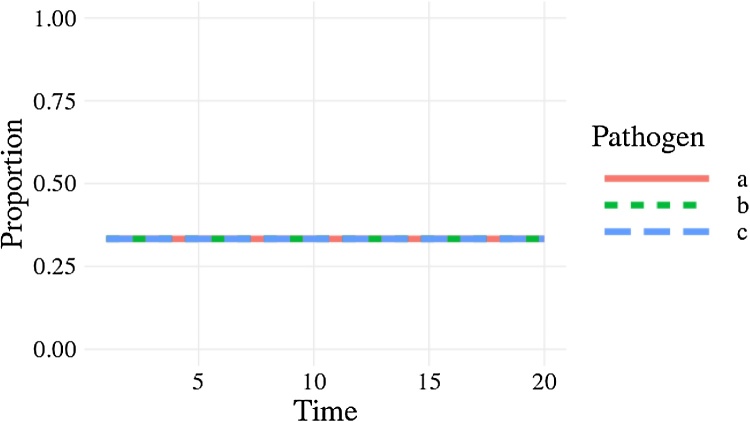
The proportion of each pathogen through time as given by Code 1. Each pathogen is a different line but are overplotted. The proportions of each pathogen do not stochastically drift as we would expect. A line plot showing the proportion of the three pathogens through time. The proportion of each pathogen in the population remains constant at 0.333.

Usually we would make some output plots to explore if our code is performing sensibly. Plotting the time course of which pathogen is infecting one individual, shows repeated infection with different pathogens as expected (Fig. 2). However, if we plot the proportion of each pathogen (Fig. 3) we quickly see that instead of stochastically varying, the proportions are identical through time and so there must be a bug present. This simple example demonstrates, first, that bugs can be subtle. Second, it is not easy to notice an error, even in just 7 lines of code. Third, it is much easier to debug code when you know there is a bug. Fourth, while plotting simulation runs is an excellent way to check model behaviour, if we had only relied on Fig. 2 we would have missed the bug. Additionally, manually checking plots is a time consuming and non-scalable method because a human has to perform this scan every test run. In summary this *ad hoc* plotting approach reduces the chances that we will catch all bugs.

**Fig. 4:**
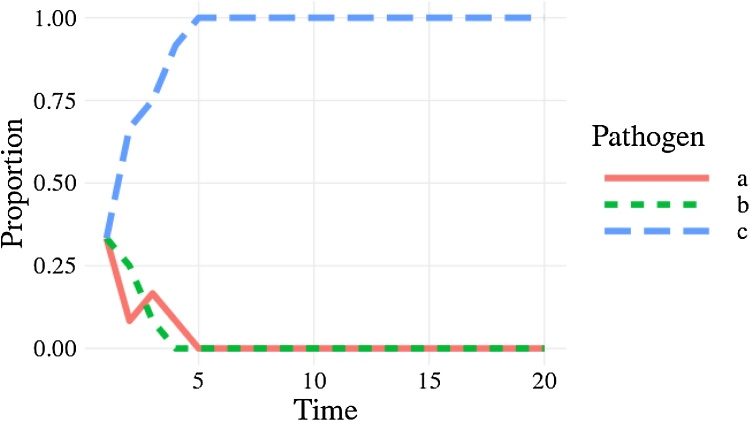
The correct behaviour with the proportion of each pathogen, as a different line, drifting to dominance or extinction. A line plot showing the proportion of the three pathogens through time. After 5 time steps, pathogen a has risen to a proportion of 1 while pathogens a and b have a proportion of 0.

The cause of the bug is that sample() defaults to sampling without replacement sample(…, replace = FALSE); this means everyone transmits their infection pathogen on a one-to-one basis rather than one-to-many as required by the model. Setting replace = TRUE fixes this (Code 2) and when we plot the proportion of each pathogen (Fig. 4) we see the correct behaviour (a single pathogen drifting to dominance). From this point there are no further bugs in the code. In the subsequent sections we will develop this base example as we consider different concepts in unit testing, resulting in well-tested code by the end. Despite there being no further bugs, we can examine how unit testing allows us to protect against misuse of the code and reinforce our confidence in the code.

Code 2: Corrected base example.

**Figure:**
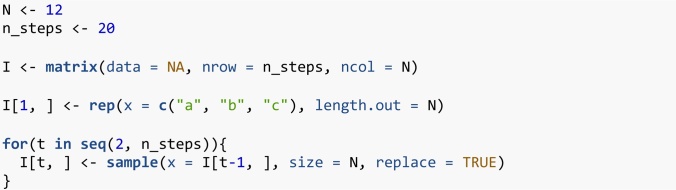

## 4 Basic unit testing

### 4.1 Write small functions

To ensure the unit tests are evaluating the exact code as run in the analysis, code should be structured in functions, which can be used both to run unit tests and to generate results as part of a larger model codebase. Make your functions compact with a single clearly-defined task. We have defined a function, initialisePop(), to initialise the population and another, updatePop(), to run one iteration of the simulation (Code 3). Organising the codebase into these bite-sized operations makes following the programming flow easier as well as understanding the code structure. Furthermore, it facilitates other good programming practices such as defensive programming and documentation — defensive programming, such as checking the class and dimensions of inputs in the first few lines of a function, ensures that the code will fail quickly and informatively if incorrect inputs are used. At this stage we have also enabled the varying of the number of pathogens using the pathogens argument in the initialisePop() function. The first iteration of the simulation, I[1,], is initialised with a repeating sequence of letters.

Code 3: Organising code into small functions.

**Figure:**
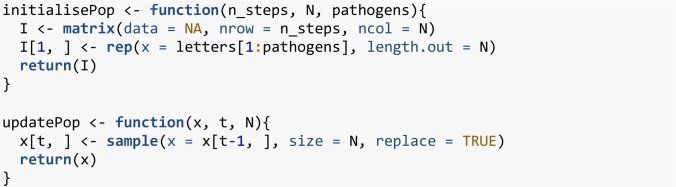

### 4.2 Test simple cases first

If we start with a small population with few pathogens, we can then easily work out exactly what the initialised population should look like (Code 4). When we initialise a population with three individuals and three pathogens, we will get the sequence as seen in the first test. When the number of individuals is greater than the number of pathogens, the sequence will be repeated as seen in the second test. Finally, when the number of individuals is greater than, but not a multiple of, the number of pathogens, the sequence will have an incomplete repeat at the end as seen in Code 4. In this sequence of tests, we have taken our understanding of the function logic, and used it to make predictions of what the results should be. We then test that the result is as expected and if everything is correct the code will return no output.

Code 4: Using simple parameter sets we can work out, beforehand, what results to expect.

**Figure:**
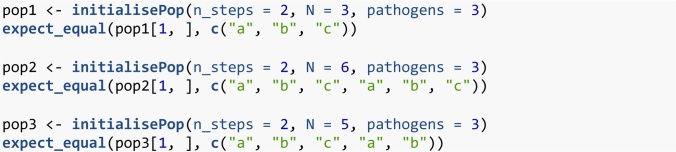

In contrast, if we had defined the initialisePop() function incorrectly, the test would fail and return an error.

Code 5: If our code is incorrect, the test will return an error.

**Figure:**
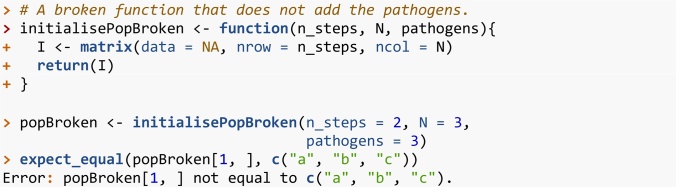

### 4.3 Test all arguments

initialisePop() has three arguments to check. First we initialise the population, and then alter each argument in turn (Code 6). Arguments n_steps and N directly change the expected dimension of the returned matrix, so we check that the output of the function is the expected size. For the pathogens argument we test that the number of pathogens is equal to the number requested.

Code 6: Test all function arguments in turn.

**Figure:**
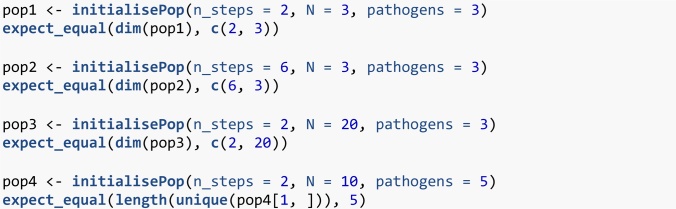

### 4.4 Does the function logic meet your expectations?

We can also cover cases that expose deviations from the logical structure of the system. After initialising the population, we expect all the rows other than the first to contain NAs. We also expect each of the pathogens a, b and c to occur at least once on the first row if pathogens = 3 and N ≥3. Finally, updatePop() performs a single simulation time step, so we expect only one additional row to be populated. Instead of testing by their numerical values, we verify logical statements of the results within our macro understanding of the model system (Code 7).

Code 7: Test more complex cases using your understanding of the system.

**Figure:**
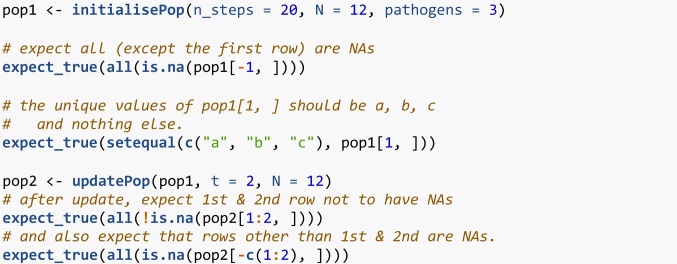

### 4.5 Combine simple functions and test them at a higher level

In the end an entire model only runs when its functions work together seamlessly. So we next check their connections; achieved through nesting functions together, or defining them at a higher level and checking the macro aspects of the model. This could be considered integration testing rather than unit testing. We define a function fullSim() that runs both initialisePop() and updatePop() to yield one complete simulation. We would expect there to be no NAs in the output from fullSim() and every element to be either a, b or c.

Code 8: Combine simple functions through nesting, to check high-level functionality.

**Figure:**
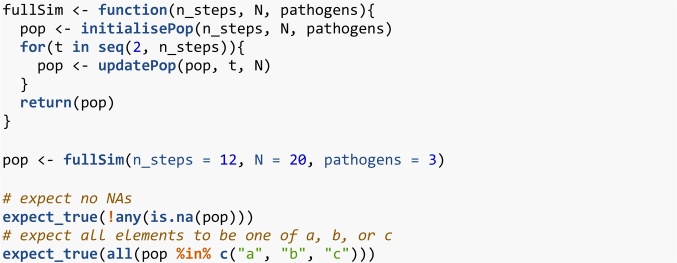

## 5 Stochastic code

Stochastic simulations are a common feature in infectious disease models. Stochastic events are difficult to test effectively because, by definition, we do not know beforehand what the result will be (Ševčíková et al., 2006; Guderlei and Mayer, 2007; Patrick et al., 2017). We can check very broad-scale properties, like Code 8, where we check the range of pathogen values. However, code could still pass and be wrong (for example the base example (Code 1) would still pass that test). There are however a number of approaches that can help.

### 5.1 Split stochastic and deterministic parts

Isolate the stochastic parts of your code. For example, updatePop() performs stochastic and deterministic operations in one line (Code 3). First updatePop() stochastically samples who gets infected by whom at iteration t. Then it takes those infection events and assigns the new infectious status for each individual. We demonstrate in Code 9 how this could be split. We accept this is a fairly exaggerated example and that splitting a single line of code into two functions is rare. The more common scenario is splitting a multi-line function into smaller functions which also brings benefits of code organisation so it does not feel like extra effort.

Code 9: Isolation of the deterministic and stochastic elements.

**Figure:**
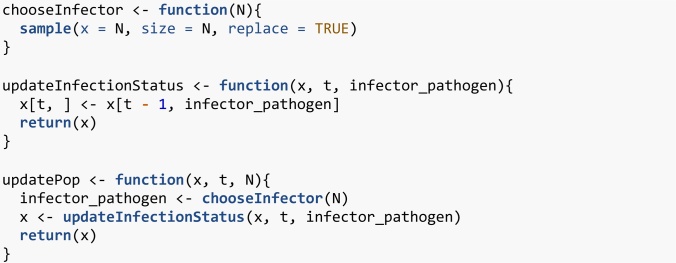

Now, half of updatePop() is deterministic so can be checked as previously discussed. We still have chooseInfector() that is irreducibly stochastic. We now examine some techniques for directly testing these stochastic parts.

### 5.2 Pick a smart parameter for a deterministic result

In the same way that we used simple parameters values in Code 4, we can often find simple cases for which stochastic functions become deterministic. For example, $X∼\text{Bernoulli}\left(p\right)$ will always generate zeroes for p=0 or ones for p=1. In the case of a single pathogen (Code 10), the model is no longer stochastic. So initialisation with one pathogen means the second time step should equal the first.

Code 10: A stochastic function can output deterministically if you can find the right parameter set.

**Figure:**
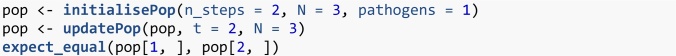

### 5.3 Test all possible answers (if few)

Working again with a simple parameter set, there are some cases where the code is stochastic, but with a small, finite set of outputs. So we can run the function exhaustively and check it returns all of the possible outputs. For a population of two people, chooseInfector() returns a length-2 vector with the possible elements of 1 or 2. There are four possibilities when drawing who is infected by whom. Both individuals can be infected by individual 1, giving the vector {1, 1}. Both individuals can be infected by individual 2, giving {2, 2}. Both individuals can infect themselves, giving {1, 2}. Or finally both individuals can infect each other, giving {2, 1}. In (Code 11), chooseInfector(N = 2) returns a length-2 vector with the indices of the infector for each infectee. paste0() then turns this length-2 vector into a length-1 string with two characters; we expect this to be one of “11”, “22”, “12” or “21”. replicate() runs the expression 300 times.

Code 11: Testing stochastic output when it only covers a few finite values.

**Figure:**
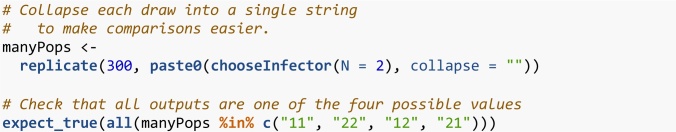

### 5.4 Use very large samples for the stochastic part

Testing can be made easier by using very large numbers. This typically involves large sample sizes or numbers of stochastic runs. For example, the clearest test to distinguish between our original, buggy code (Code 1) and our correct code (Code 2) is that in the correct code there is the possibility for an individual to infect more than one individual in a single time step. In any given run this is never guaranteed, but the larger the population size the more likely it is to occur. With a population of 1000, the probability that no individual infects two others is vanishingly rare (Code 12). As this test is now stochastic we should set the seed, using set.seed(), of the random number generator to make the test reproducible.

Code 12: Testing that the code does allow one individual to infect multiple individuals.

**Figure:**
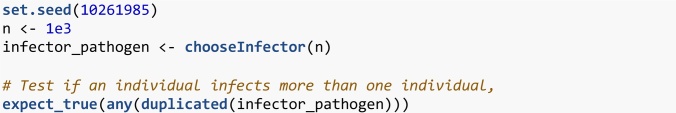

If we have an event that we know should never happen, we can use a large number of simulations to provide stronger evidence that it does not stochastically occur. However, it can be difficult to determine how many times is reasonable to run a simulation, especially if time is short. This strategy works best when we have a specific bug that occurs relatively frequently (perhaps once every ten simulations or so). If the bug occurs every ten simulations and we have not fixed it, we would be confident that it will occur at least once if we run the simulation 500 or 1000 times. Conversely, if the bug does not occur even once in 500 or 1000 simulations, we can be fairly sure we have fixed it. Similarly, a bug might cause an event that should be rare to instead occur very regularly or even every time the code is run. In our original buggy code (Code 1) we found that the proportions remained identical for entire simulations. We would expect this to happen only very rarely. We can run a large number of short simulations to check that this specific bug is not still occurring by confirming that the proportion of each pathogen is not always the same between the first and last time point. As long as we find at least one simulation where the proportions of each pathogen are different between the first and last iteration, we can be more confident that the bug has been fixed.

Code 13: Assessing if a bug fix was a likely success with large code runs, when the bug was appearing relatively frequently.

**Figure:**
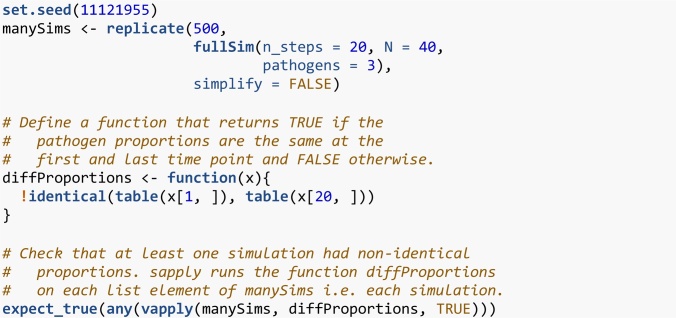

This example can be thought of more generally as having an expected distribution of an output and using a statistical test to compare the simulation results with the expectation. Here, we had a binomial event (was the pathogen proportions different between the first and last time step) and an expected frequency of this event (greater than zero). This approach to testing stochastic code is detailed in Ševčíková et al. (2006); Guderlei and Mayer (2007) and Patrick et al. (2017). If we know a property of the expected distribution (the mean for example) we can run the simulation repeatedly and use a statistical test to compare the distribution of simulated outputs to the expected distribution.

## 6 Further testing

### 6.1 Test incorrect inputs

As well as testing that functions work when given the correct inputs, we should also test that they behave sensibly when given the wrong ones. This typically involves the user inputting argument values that do not make sense. This may be, for example, because the inputted argument values are the wrong class, in the wrong numeric range, or have missing data values — therefore it is useful to test that functions fail gracefully. This is especially true for external, exported functions, available to a user on a package’s front-end. However, it is not always obvious what constitutes an ‘incorrect value’ even to the person who wrote the code. In some cases, inputting incorrect argument values may cause the function to fail quickly. In other cases, code may run silently giving false results or take a long time to throw an error. Both of these cases can be serious or annoying and difficult to debug afterwards. In this section we identify frailties in the code that are conceptually different to a bug; the model as specified is already implemented correctly. Instead we are making the code more user-friendly and user-safe to protect against mistakes during future code re-use. Often for these cases, the expected behaviour of the function should be to give an error. There is no correct output for an epidemiological model with -1 pathogens. Instead the function should give an informative error message. Often the simplest solution is to use defensive programming and include argument checks at the beginning of functions. We then have to write slightly unintuitive tests for an expression where the expected behaviour is an error. If the expression does not throw an error, the test should throw an error (as this is not the expected behaviour). Conversely, if the expression does throw an error, the test should pass and not error. We can use the expect_error() function for this task. This function takes an expression as its first argument and reports an error if the given expression does not throw an expected error. We can first check that the code sensibly handles the user inputting a string instead of an integer for the number of pathogens. Because this expression throws an error, expect_error() does not error and the test passes.

Code 14: Testing incorrect pathogen inputs.

**Figure:**
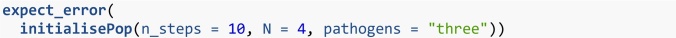

Now we contrast what happens if the user inputs a vector of pathogens to the initialisePop() function. Here we imagine the user intended to run a simulation with three pathogens: 1, 2 and 3.

Code 15: A failing test for incorrect pathogen inputs.

**Figure:**
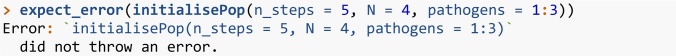

This test fails because the function does not throw an error. Instead the code takes the first element of pathogens and ignores the rest. Therefore, a population is created with one pathogen, not three, which is almost certainly not what the user wanted. Here, the safest fix is to add an explicit argument check at the top of the function (Code 16). The same test now passes because initialisePop() throws an error when a vector is supplied to the pathogens argument.

Code 16: New definition, using defensive programming, of the initialisePop() function and a passing test for incorrect pathogen inputs.

**Figure:**
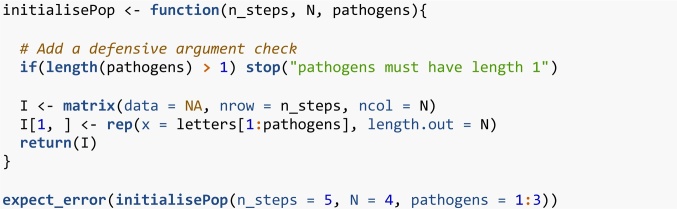

We can similarly check how the code handles a user inputting a vector of numbers to the n_steps argument (perhaps thinking it needed a vector of all time points to run). In Code 16, initialisePop() does not throw an error if a vector is supplied to n_steps. However, fullSim() does throw an error if a vector is supplied to n_steps. The error message is “Error in seq.default(2, n_steps) : ‘to’ must be of length 1”. While it is a good thing that fullSim() throws an error, this error message is not very informative. If the code that runs before the error is thrown (in this case the initialisePop() function) takes a long time, it can also be time consuming to work out what threw the error (though the debug() function can help). It is also a signature of fragile code that the error is coincidental; a small change in the code might stop the error from occurring. These considerations all point towards defensive programming as a good solution. We can add an additional argument check to initialisePop(). Importantly, we then want to check that fullSim() errors in the correct place (i.e. in initialisePop() rather than afterwards). We can achieve this using the regexp argument of expect_error() that compares the actual error message to the expected error messages. The test will only pass if the error message contains the string provided.

Code 17: Another new definition of the initialisePop() function and a passing test for the fullSim() function.

**Figure:**
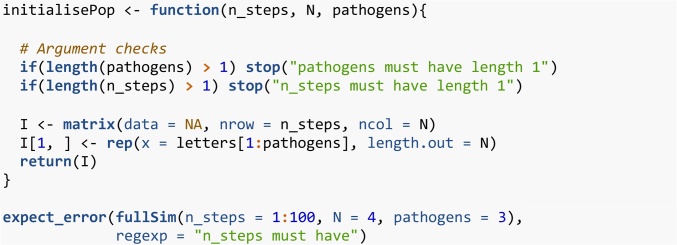

### 6.2 Test special cases

When writing tests it is easy to focus on standard behaviour. However, bugs often occur in *special cases*—when the code behaves qualitatively differently at a certain value or certain combinations of parameter values. For example, in *R*, selecting two or more columns from a matrix e.g. my_matrix[, 2:3] returns a matrix while selecting one column e.g. my_matrix[, 2] returns a vector. Code that relies on the returned object being a matrix would fail in this special case. These special cases can often be triggered with parameter sets that are at the edge of parameter space. This is where an understanding of the functional form of the model can help. Consider a function divide(x, y) that divides x by y. We could test this function by noting that y * divide(x, y) should return x. If we write code that tests standard values of x and y such as (2 * divide(3, 2)) = = 3 we would believe the function works for nearly all values of division, unless we ever try y = 0. We checked earlier if the n_steps argument of initialisePop() worked by verifying that the returned population had the correct dimensions (Code 6). We can test the fullSim() function in the same way (Code 18). However, if we try to run the same test with n_steps = 1 we get an error before we even get to the test.

Code 18: fullSim() does not give a population matrix with the correct number of rows if one iteration is requested.

**Figure:**
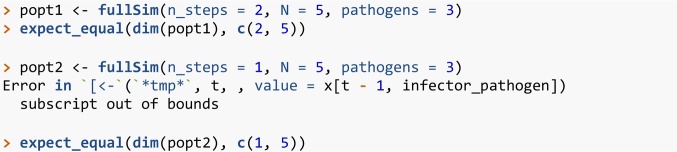

In general, requesting a simulation with 1 time step is not without epidemiological meaning and is not an objectively wrong use of the function. The code behaves qualitatively differently for n_steps = 1 than for n_steps = 2 because the loop in Code 8 runs from 2 to n_steps setting t to each value in turn. When n_steps is 2 or more, this follows the sequence {2,3,...}. When n_steps is 1, this instead follows the sequence {2,1}. The population is initialised with 1 row but the code still tries to store the results in the second row of the population matrix. For special cases like this, it may be rather subjective what the correct behaviour should be. It might be appropriate to simply declare that this parameter value is not allowed. This should be stated in the documentation and the function should throw an error. Here however, we will decide that this is a valid parameter value. We should change the code to handle this special case, and use the new test to check that our new code works. In Code 19 we use an if statement to explicitly handle the special case of n_steps = 1 and our test now passes.

Code 19: Handle the special case of t = 1 and test the new code.

**Figure:**
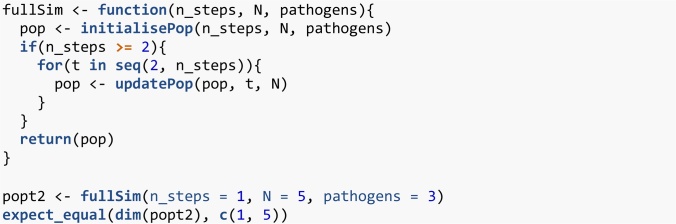

### 6.3 Unit testing frameworks

Most programming languages have established testing packages. For *R*, there is the testthat package as already discussed as well as other packages such as tinytest which has the benefit of having no dependencies. When structuring *R* code as standalone scripts, the tests should be saved in one or more scripts either in the same directory as the scripts in which the functions are defined, or within a subdirectory of the same project directory. The testing script needs to load all the functions it is going to test (with source() for example) and then run the tests. When structuring *R* code as a package, tests should be kept in the directory tests/testthat; further requirements to the structure can be found in Chapter 7 of Wickham (2015). All the tests in a package can then be run with test() from the devtools package (Wickham et al., 2019) or check() for additional checks relevant to the package build. If the code is to be part of a package then these tools are essential to run the code within the context of a build environment. These tools also provide a clean environment to highlight if a test was previously relying on objects defined outside of the test script. Furthermore, organising code in a package provides further benefits such as tools to aid the writing of documentation, systematic handling of dependencies, and tools to track whether every line of code in the package is tested such as the covr package (Hester, 2020). The practice of organising code as a package, even if there is no expectation that others will use the code, is underappreciated and underused in epidemiology. The testing frameworks of other programming languages differ but the main concept of comparing a function evaluation to the expected output remains the same. In *Julia* there is the Test package (Bezanson et al., 2017b). The basic structure for tests with this package is shown in Code 20. We name the test and write a single expression that evaluates to TRUE or FALSE. For a *Julia* package, unit tests reside in test/runtests.jl and tests are run with Pkg.test().

Code 20: Julia test example.

**Figure:**
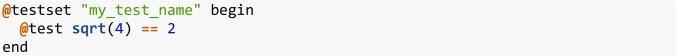

Finally, in *Python* tests can be performed using the unittest framework (Python Core Team, 2015b); tests must be written into a class that inherits from the TestCase class. The tests must be written as methods with self as the first argument. An example test script is shown in Code 21. Tests should be kept in a directory called Lib/test, and the filename of every file with tests should begin with “test_”.

Code 21: Python test example.

**Figure:**
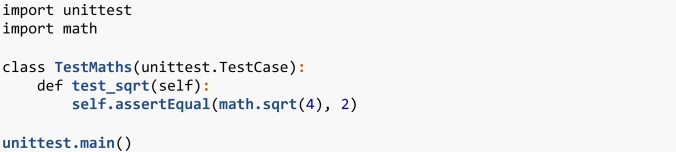

## 7 Continuous integration

If your code is under version control (Osborne et al., 2014; Wilson et al., 2014) and hosted online (e.g. on GitHub, GitLab or BitBucket), you can automate the running of unit tests—also known as *continuous integration*. In this setup, whenever you push code changes from your local computer to the online repository, any tests that you have defined get run automatically. Furthermore, these tests can be automated to run periodically so that bugs caused by changes in dependencies are flagged. There are various continuous integration services such as www.travis-ci.org, GitHub actions and GitLab pipelines. These services are often free on a limited basis, or free if your code is open source. We briefly describe the setup of the simplest case using Travis CI. Setting up continuous integration is very straightforward for *R* code already organised into a package and hosted openly on GitHub. Within your version-controlled folder that contains the *R* code, you add a one-liner file named “.travis.yml” that contains a description of which language the code uses.

Code 22: A basic travis yml file.

**Figure:**
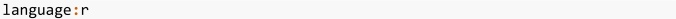

This file can also be created with use_travis() from the usethis package. You then sign up to travis-ci.org and point it to the correct GitHub repository. To authenticate and trigger the first automatic test you need to make a minor change to your code, commit and push to GitHub. More details on setting up Travis, and how to continuously track test coverage using covr and codecov, can be found in Chapter 14.3 of Wickham (2015).

## 8 Concluding remarks

It is vital that infectious disease models are coded to minimise bugs. Unit testing is a well-defined, principled way to do this. There are many frameworks that make aspects of unit testing automatic and more informative and these should be used where possible. The basic principles of unit testing are simple but writing good tests is a skill that takes time, practice and thought. However, ensuring your code is robust and well-tested saves time and effort in the long run and helps prevent spurious results. Our aim in this paper was to demonstrate tailored results for infectious disease modelling. There are a number of standard programming approaches to unit testing which would be good further reading (Chapter 7 of Wickham (2015), Osborne et al. (2014); Wilson et al. (2014)). As demonstrated here, it is initially time-consuming to program in this way, but over time it becomes habitual, and both you and the policy-makers who use your models will benefit from it.

## Code availability

Please see the fully-reproducible and version-controlled code at www.github.com/timcdlucas/unit_test_for_infectious_disease or the archived version at https://doi.org/10.5281/zenodo.4293667.

## Funding sources

TMP, TDH, TCDL and ELD gratefully acknowledge funding of the NTD Modelling Consortium by the Bill & Melinda Gates Foundation (BMGF) (grant number OPP1184344). Views, opinions, assumptions or any other information set out in this article should not be attributed to BMGF or any person connected with them. TMP’s PhD is supported by the Engineering & Physical Sciences Research Council, Medical Research Council and the University of Warwick (grant number EP/L015374/1). TMP thanks the Big Data Institute for hosting him during this work. All funders had no role in the study design, collection, analysis, interpretation of data, writing of the report, or decision to submit the manuscript for publication.

## CRediT authorship contribution statement

**Tim CD Lucas:** Conceptualisation, Data curation, Formal analysis, Investigation, Methodology, Project administration, Software, Visualisation, Writing - original draft, Writing - review & editing. **Timothy M Pollington:** Conceptualisation, Software, Validation, Visualisation, Writing - review & editing. **Emma L Davis:** Writing - review & editing. **T Déirdre Hollingsworth:** Funding acquisition, Writing - review & editing. The authors would like to thank three anonymous reviewers for their useful comments.

## Declaration of Competing Interest

The authors have no competing interests.

## References

[bib0005] American Society of Clinical Oncology (2016). Retraction. J. Clin. Oncol..

[bib0010] Baker S. (2020). Pandemic Response Shines Spotlight on Coding in Science. https://www.timeshighereducation.com/news/pandemic-response-shines-spotlight-coding-science.

[bib0015] BCS, The Chartered Institute for IT (2020). Professionalising Software Development in Scientific Research. https://www.bcs.org/media/5780/professionalising-software-development.pdf.

[bib0020] Behrend M.R., Basáñez M.-G., Hamley J., Porco T.C., Stolk W.A., Walker M., de Vlas S.J., NTD Modelling Consortium (2020). Modelling for policy: the five principles of the Neglected Tropical Diseases Modelling Consortium. PLoS Negl. Trop. Dis..

[bib0025] Bezanson J., Edelman A., Karpinski S., Shah V.B. (2017). *Julia*: a fresh approach to numerical computing. SIAM Rev..

[bib0030] Bezanson J., Edelman A., Karpinski S., Shah V.B., other contributors (2017). Test V1.4.1: Simple Unit Testing Functionality in Julia. https://github.com/JuliaLang/julia/blob/master/stdlib/Test/src/Test.jl.

[bib0035] Mishap Investigation Board (1999). Mars Climate Orbiter Mishap Investigation Board Phase I Report November 10, 1999.

[bib0040] Chawla D.S. (2020). Critiqued Coronavirus Simulation Gets Thumbs up From Code-checking Efforts. https://www.nature.com/articles/d41586-020-01685-y.

[bib0045] Csardi G., FitzJohn R., Jombart T., Kamvar Z.N., Ross N. (2020). RECON Guidelines. Best Practices for Package Development. https://www.repidemicsconsortium.org/resources/guidelines/.

[bib0050] Den Boon S., Jit M., Brisson M., Medley G., Beutels P., White R., Flasche S. (2019). Guidelines for multi-model comparisons of the impact of infectious disease interventions. BMC Med..

[bib0055] Eglen S.J. (2020). CODECHECK Certificate for Paper: Report 9: Impact of Non-Pharmaceutical Interventions (NPIs) to Reduce COVID-19 Mortality and Healthcare Demand. March 16, 2020.

[bib0060] Ferguson N.M. (2020). Tweet From @neil_ferguson. https://twitter.com/neil_ferguson/status/1241835454707699713.

[bib0065] Ferguson N.M., MRC Centre for Global Infectious Disease Analysis (2020). Covid-sim. https://github.com/mrc-ide/covid-sim.

[bib0070] Ferguson N.M., Cummings D.A.T., Fraser C., Cajka J.C., Cooley P.C., Burke D.S. (2006). Strategies for mitigating an influenza pandemic. Nature.

[bib0075] Ferguson N.M., Laydon D., Nedjati-Gilani G., Imai N., Ainslie K., Baguelin M., Bhatia S. (2020). Impact of Non-Pharmaceutical Interventions (NPIs) to Reduce COVID-19 Mortality and Healthcare Demand. https://www.imperial.ac.uk/mrc-global-infectious-disease-analysis/covid-19/report-9-impact-of-npis-on-covid-19/.

[bib0080] Guderlei R., Mayer J. (2007). Statistical metamorphic testing testing programs with random output by means of statistical hypothesis tests and metamorphic testing. Seventh International Conference on Quality Software (Qsic 2007).

[bib0085] Heesterbeek H., Anderson R.M., Andreasen V., Bansal S., De Angelis D., Dye C., Eames K.T.D. (2015). Modeling infectious disease dynamics in the complex landscape of global health. Science.

[bib0090] Hellewell J., Abbott S., Gimma A., Bosse N.I., Jarvis C.I., Russell T.W., Munday J.D. (2020). Feasibility of controlling COVID-19 outbreaks by isolation of cases and contacts. Lancet.

[bib0095] Herndon T., Ash M., Pollin R. (2014). Does High Public Debt Consistently Stifle Economic Growth? A Critique of Reinhart and Rogoff.”. Cambridge J. Econ..

[bib0100] Hester J. (2020). Covr: Test Coverage for Packages. https://CRAN.R-project.org/package=covr.

[bib0105] Hollingsworth T.D., Medley G.F. (2017). Learning from Multi-Model Comparisons: Collaboration Leads to Insights, but Limitations Remain. Epidemics.

[bib0110] IHME COVID-19 health service utilization forecasting team, Murray C.J.L. (2020). Forecasting COVID-19 Impact on Hospital Bed-Days, ICU-days, Ventilator Days and Deaths by US State in the Next 4 Months. medRxiv.

[bib0115] Jenness S.M., Goodreau S.M., Morris M. (2018). EpiModel: an R package for mathematical modeling of infectious disease over networks. J. Stat. Softw..

[bib0120] NASA (2020). Mariner 1. https://nssdc.gsfc.nasa.gov/nmc/spacecraft/display.action?id=MARIN1.

[bib0125] Neupane B.J., Neupane R.P., Luo Y., Yoshida W.Y., Sun R., Williams P.G. (2019). Characterization of Leptazolines A–D, Polar Oxazolines from the Cyanobacterium Leptolyngbya Sp., Reveals a Glitch with the ‘Willoughby–Hoye’ Scripts for Calculating NMR Chemical Shifts. Org. Lett..

[bib0130] Osborne J.M., Bernabeu M.O., Bruna M., Calderhead B., Cooper J., Dalchau N., Dunn S.-J. (2014). Ten simple rules for effective computational research. PLoS Comput. Biol..

[bib0135] Patrick M., Donnelly R., Gilligan C.A. (2017). A toolkit for testing stochastic simulations against statistical oracles. 2017 IEEE International Conference on Software Testing, Verification and Validation (ICST).

[bib0140] Python Core Team (2015). *Python V3.8.2: A Dynamic, Open Source Programming Language*. Python Software Foundation. https://www.python.org/.

[bib0145] Python Core Team (2015). Unittest V3.8.2: Unit Testing Framework. https://docs.python.org/3/library/unittest.html.

[bib0150] R Core Team (2018). R V3.6.3: A Language and Environment for Statistical Computing. https://www.R-project.org/.

[bib0155] Santos B.O., Fernando S.M. (2020). EpiDynamics: Dynamic Models in Epidemiology. https://CRAN.R-project.org/package=EpiDynamics.

[bib0160] Ševčíková H., Borning A., Socha D., Bleek W.-G. (2006). Automated testing of stochastic systems: a statistically grounded approach. Proceedings of the 2006 International Symposium on Software Testing and Analysis.

[bib0165] SPI-M (2020). Scientific Pandemic Influenza Group on Modelling (Spi-M). https://www.gov.uk/government/groups/scientific-pandemic-influenza-subgroup-on-modelling.

[bib0170] Wickham H. (2011). Testthat V.2.3.2: get started with testing. R J..

[bib0175] Wickham H. (2015). O’Reilly Media, Inc.

[bib0180] Wickham H. (2019). Advanced R First Edition. http://adv-r.had.co.nz/Exceptions-Debugging.html.

[bib0185] Wickham H., Hester J., Chang W., Studio R., R Core Team (2019). Devtools V2.3.0: Tools to Make Developing R Packages Easier. https://CRAN.R-project.org/package=devtools.

[bib0190] Willem L., Verelst F., Bilcke J., Hens N., Beutels P. (2017). Lessons from a decade of individual-based models for infectious disease transmission: a systematic review (2006-2015). BMC Infect. Dis..

[bib0195] Wilson G., Aruliah D.A., Titus B.C., Chue H.N.P., Davis M., Guy R.T., Haddock S.H.D. (2014). Best practices for scientific computing. PLoS Biol..

