# Supplementary material for: Responsible modelling: Unit testing for infectious disease epidemiology
Source: Epidemics. 2020 Dec;33:100425. doi: 10.1016/j.epidem.2020.100425 (PMC7690327; doi:10.1016/j.epidem.2020.100425)
Supplement: Supplementary file 1 [file mmc1.pdf]

Responsible modelling: Unit testing for infectious disease epidemiology.

Tim CD Lucas, Timothy M Pollington, Emma L Davis and T Déirdre Hollingsworth

Contents

1 Load necessary packages **1**

2 A more realistic modelling example **1**

3 Basic unit testing **4**
Write small functions . . . 4
Test simple cases first . . . 6
Test all arguments . . . 7
Does the function logic meet your expectations? . . . 7
Combine simple functions and test them at a higher-level . . . 8

4 Stochastic code **8**
Split stochastic and deterministic parts . . . 9
Pick a smart parameter for a deterministic result . . . 10
Test all possible answers (if few) . . . 11
Use very large samples for the stochastic part . . . 11

5 Further testing **12**
Test incorrect inputs . . . 12
Test special cases . . . 13
5.1 Session Info . . . 15
References . . . 16

1 Load necessary packages

```
library(ggplot2)
library(testthat)
library(reshape2)
```

2 A more realistic modelling example

Here we define a simple model that is less contrived compared to the main text. We again demonstrate how to effectively write unit tests for it in *R* code. We aim to follow the same structure as in the main text but in this example we do not need to worry about the code being short.

We will implement a stochastic, continuous time, SIR model using code from EpiRecipes (Frost 2020) as a starting point. The number of individuals in the three classes, susceptible, infectious and recovered, are

described by the equations

$$\frac{dS}{dt} = -\beta SI \tag{1}$$

$$\frac{dI}{dt} = \beta SI - \gamma I \tag{2}$$

$$\frac{dR}{dt} = \gamma I \tag{3}$$

In order to simulate continuous time dynamics we use the Gillespie algorithm. In each iteration of the model, exactly one event happens, either an infection or a recovery. The time step in between events is a continuous, positive number that is drawn from an exponential distribution with a rate equal to the sum of two event rates (i.e. infection and recovery).

Code S1: Base example of the multi-pathogen re-infection model.

```
# set the seed so that our simulations are
#   reproducible.
set.seed(20200908)

# Define parameters
beta <- 0.001 # Transmsission parameter
gamma <- 0.4 # Recovery parameter
N <- 1000 # Population size
S0 <- 990 # Starting number of susceptibles
I0 <- 10 # Starting number of infected
R0 <- N - S0 - I0 # Starting number of Recovered
tf <- 2000 # Run until time reaches this value

# Initialise the simulation.
time <- 0
S <- S0
I <- I0
R <- R0
ta <- numeric(0)
Sa <- numeric(0)
Ia <- numeric(0)
Ra <- numeric(0)

# Run the simulation until time reaches
#   tf.
while (time < tf) {

    ta <- c(ta, time)
    Sa <- c(Sa, S)
    Ia <- c(Ia, I)
    Ra <- c(Ra, R)

    # Infection rate.
    pf1 <- beta * S * I
    # Transmission rate
    pf2 <- gamma * I
    # Total rate of all events
    pf <- pf1 + pf2
    # Draw waiting time for this event.
```

```
    dt <- rexp(1, rate = pf)
    # Increment the current time.
    time <- time + dt

    # If we have reach time tf, finish the simulation
    if (time > tf) {
        break
    }

    # Select whether the event is a transmission
    #   or recovery event.
    if (runif(1) < (pf1 / pf)) {
        # Transmission event.
        S <- S - 1
        I <- I + 1
    } else {
        # Recovery event
        I <- I - 1
        R <- R + 1
    }

    # If the epidemic is extinct, end the simulation
    if (I == 0) {
        break
    }
}

results <- data.frame(time = ta, S = Sa, I = Ia, R = Ra)
```

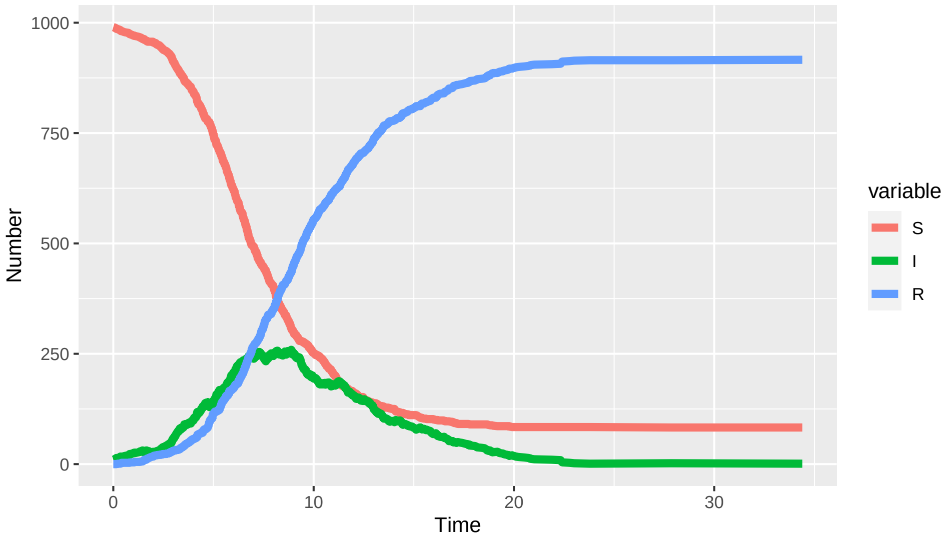

Figure S1: Example simulation output from the original code.

3 Basic unit testing

Write small functions

To ensure the unit tests are evaluating the exact code as run in the analysis, code should be structured in functions, which can be used to both run unit tests with and to generate results as part of a larger model codebase. Make your functions compact with a single clearly-defined task. We have split the code into a number of functions that do relatively well defined tasks such as initialising the simulation, handling a single transmission event or a single recovery event as well as a large function (`runSim()`) that combines these functions. (Code S2).

Code S2: Organising code into small functions.

```
initialiseSim <- function(S0, I0, R0){
  sim <- list(
    time = 0,
    S = S0,
    I = I0,
    R = R0,
    ta = numeric(0),
    Sa = numeric(0),
    Ia = numeric(0),
    Ra = numeric(0)
  )
  return(sim)
}

addNew <- function(sim){
  sim$ta <- c(sim$ta, sim$time)
  sim$Sa <- c(sim$Sa, sim$S)
  sim$Ia <- c(sim$Ia, sim$I)
  sim$Ra <- c(sim$Ra, sim$R)
  return(sim)
}

infection <- function(sim){
  sim$S <- sim$S - 1
  sim$I <- sim$I + 1
  return(sim)
}

recovery <- function(sim){
  sim$I <- sim$I - 1
  sim$R <- sim$R + 1
  return(sim)
}

nextEvent <- function(sim, pf1, pf){
  # Select whether the event is a transmission
  #   or recovery event.
  if (runif(1) < (pf1 / pf)) {
    # Transmission event.
    sim <- infection(sim)
  } else {
    # Recovery event
```

```r
    sim <- recovery(sim)
  }
  return(sim)
}

calcRates <- function(sim, beta, gamma){
  rates <- list()
  # Infection rate.
  rates$pf1 <- beta * sim$S * sim$I
  # Transmission rate
  rates$pf2 <- gamma * sim$I
  # Total rate of all events
  rates$pf <- rates$pf1 + rates$pf2
  # Draw waiting time for this event.
  rates$dt <- rexp(1, rate = rates$pf)
  return(rates)
}



runSim <- function(sim, beta, gamma, tf){
  # Run the simulation until time reaches tf.
  while (sim$time < tf) {

      sim <- addNew(sim)

      rates <- calcRates(sim, beta, gamma)

      # Increment the current time.
      sim$time <- sim$time + rates$dt

      # If we have reached time tf, finish the simulation
      if (sim$time > tf) {
          break
      }

      # Do the next event.
      sim <- nextEvent(sim, rates$pf1, rates$pf)

      # If the epidemic is extinct, end the simulation
      if (sim$I == 0) {
          break
      }
  }
  results <- data.frame(time = sim$ta, S = sim$Sa, I = sim$Ia, R = sim$Ra)
  return(results)
}
```

As an informal first check we can plot a single simulation and check that it gives similar results to the earlier plot and behaves broadly how we would expect an SIR model to behave. For example, we expect the number of infectious individuals to increase and then decrease again while we expect the susceptible and recovered classes to monotonically decrease and increase respectively.

Code S3: Run a full simulation with the code organised in functions.

```
sim <- initialiseSim(S0, I0, R0)

simulation <- runSim(sim, beta, gamma, tf)

sir_out_long <- melt(simulation, "time")
ggplot(sir_out_long,
       aes(x = time, y = value, colour = variable, group = variable))+
  geom_line(lwd = 2) +
  xlab("Time") + ylab("Number")
```

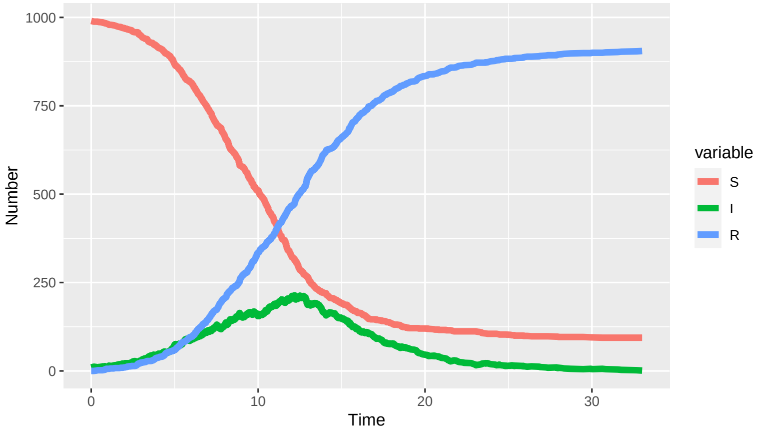

Figure S2: Example simulation output from the refactored code.

Test simple cases first

As there are quite a few functions already in our code we will not write unit tests for all of them here. However, in Code S4 we will write some tests for simple aspects of the code. For example we can confirm that the `infection()` function works as expected. We expect this function to increase the number of infectious individuals by one, decrease the number of susceptible individuals by one and leave the rest of the object unchanged. If we start with 1 susceptible and 0 infectious or recovered individuals, this function should result in 0 susceptible individuals, 1 infectious individual and 0 recovered individuals. We can see that the way we have written the code has separated the process of finding out what event happens, from the book keeping of making the change once an event has been selected. With 0 infectious individuals we would not expect to ever have an infection event, but separating the functionality of the code allows us to test that the bookkeeping works without worrying about other aspects.

Code S4: Using simple parameter sets we can work out beforehand what results to expect.

```
sim1 <- initialiseSim(1, 0, 0)
sim2 <- infection(sim1)

# Check that the classes are change by the right amount.
expect_equal(sim2$S, 0)
expect_equal(sim2$I, 1)
expect_equal(sim2$R, 0)

# Check that some other elements of the object are unchanged.
expect_equal(sim2$time, sim1$time)
```

Test all arguments

`calcRates()` has three arguments to check. If we consider a baseline population with one susceptible, one infectious and zero recovered individuals, with both `beta` and `gamma` set to one we can easily work out the expected outputs. In Code S5 we will focus our tests on the two rates: the transmission rate `pf1` and the recovery rate `pf2`. Given that the SIR transmission rate is βSI and given that β, S and I are all one, `pf1` should be one. Similarly, given a recovery rate of γI and with γ set to one, `pf2` should also be one.

Code S5: Baseline before testing all function arguments in turn.

```
sim1 <- initialiseSim(S0 = 1, I0 = 1, R0 = 0)
r1 <- calcRates(sim = sim1, beta = 1, gamma = 1)

expect_equal(r1$pf1, 1)
expect_equal(r1$pf2, 1)
```

From there we can alter each of the three parameters in turn and check the behaviour is as expected (Code S6). First we will set `beta` to 2 and so we expect `pf1` to be 2 while `pf2` should not change. Second we will set `gamma` to 2 and so we expect `pf2` to be 2 while `pf1` should not change. Finally, we will initialise a new simulation with `I0 = 2` and we will then expect `pf1` and `pf2` to be equal to 2.

Code S6: Test all function arguments in turn.

```
sim1 <- initialiseSim(S0 = 1, I0 = 1, R0 = 0)

# Set beta = 2
r2 <- calcRates(sim = sim1, beta = 2, gamma = 1)

expect_equal(r2$pf1, 2)
expect_equal(r2$pf2, 1)


# Set gamma = 2
r3 <- calcRates(sim = sim1, beta = 1, gamma = 2)

expect_equal(r3$pf1, 1)
expect_equal(r3$pf2, 2)


# Set I0 = 2
sim2 <- initialiseSim(S0 = 1, I0 = 2, R0 = 0)

r4 <- calcRates(sim = sim2, beta = 1, gamma = 1)

expect_equal(r4$pf1, 2)
expect_equal(r4$pf2, 2)
```

Does the function logic meet your expectations?

We can also cover cases that expose deviations from the logical structure of the system. The function `addNew()` changes the object ready for a new iteration in the simulation. Therefore the length of a number of elements should increase by one. Instead of testing their numerical values, we verify logical statements of the results within our macro understanding of the model system (Code S7).

Code S7: Test logical structure of the code rather than the specific numerical values.

```r
sim1 <- initialiseSim(10, 5, 0)
sim2 <- addNew(sim1)

# Check that the vectors get 1 longer.
expect_equal(length(sim2$ta), length(sim1$ta) + 1)
expect_equal(length(sim2$Sa), length(sim1$Sa) + 1)

# Check that some other elements of the object are unchanged.
expect_equal(length(sim2$S), length(sim1$S))
expect_equal(length(sim2$time), length(sim1$time))
```

Combine simple functions and test them at a higher-level

In the end an entire model only runs when its functions work together seamlessly. So we next check their connections; achieved through nesting functions together, or defining them at a higher level and checking the macro aspects of the model. This step could be considered integration testing depending on how precisely you define unit testing versus integration testing. We have already defined a function `runSim()` that runs a few different smaller functions to perform one full simulation. We would expect the output from `runSim()` to be a dataframe with four columns and no `NA`s and we would expect no values of the time column to be greater than `tf`. We would also expect all of the values in the `S`, `I` and `R` columns to be integers greater than or equal to zero. These are all general properties that should be true regardless of the exact realisation of the simulation. Furthermore, checking the maximum value in the time column is, to an extent, an aggregate property that could not be tested at a lower level.

Code S8: Combine simple functions through nesting to check higher-level functionality.

```r
set.seed(13131)
sim <- initialiseSim(S0 = 900, I0 = 100, R0 = 0)

tf <- 20
simulation <- runSim(sim = sim, beta = 0.1, gamma = 0.04, tf = tf)

# Check the shape of the output
expect_true(inherits(simulation, 'data.frame'))
expect_equal(ncol(simulation), 4)

# Test for NAs
expect_true(all(!is.na(simulation)))

# Check that all but 1 time point is less than tf.
expect_true(all(simulation$time < tf))

# Check that S, I and R are positive integers.
#   R is not very strict about integers vs doubles.
#   So we instead just check that all the values are
#   very close to whole numbers.
expect_true(all(simulation[, -1] >= 0))
expect_true(all((simulation[, -1] - round(simulation[, -1])) < 1e-10))
```

4 Stochastic code

Stochastic simulations are a common feature in infectious disease models. Stochastic events are difficult to test effectively because, by definition, we do not know beforehand what the result will be. There are however

a number of approaches that can help.

Split stochastic and deterministic parts

Isolate the stochastic parts of your code. For example, `nextEvent` performs stochastic and deterministic operations (Code S9).

Code S9: Isolation of the deterministic and stochastic elements.

```
nextEvent <- function(sim, pf1, pf){
  # Stochastically select whether the event is a
  #   transmission or recovery event.
  if (runif(1) < (pf1 / pf)) {
    # Deterministically handle a
    #   transmission event.
    sim <- infection(sim)
  } else {
    # Deterministically handle a
    #   recovery event
    sim <- recovery(sim)
  }
  return(sim)
}
```

The function takes a random uniform number to determine whether an infection or recovery event takes place. It then performs that event, although we note that we have already pulled much of the code out into the two functions `infection()` and `recovery()`. However, we can split this code into two functions, one completely deterministic (`nextEvent()`), and one very small function (`chooseEvent()`) that carries out the stochastic component. The deterministic function can be tested using the guidelines above while in the following sections we will look at methods to test the stochastic function. We note that perhaps a better method here would be to make the first argument of `nextEvent()` take a random uniform number. In that case `nextEvent()` would be deterministic and the only stochastic code would be a single call to `runif()`. Given that the `runif()` function is a base *R* function that is well-tested, further testing is unnecessary. However, for demonstration purposes we have split the function as below so that we can subsequently demonstrate how to test stochastic functions.

Code S10: Isolation of the deterministic and stochastic elements.

```
chooseEvent <- function(pf1, pf){
  runif(1) < (pf1 / pf)
}

nextEvent <- function(sim, infection){
  # Select whether the event is a transmission
  #   or recovery event.
  if (infection) {
    # Transmission event.
    sim <- infection(sim)
  } else {
    # Recovery event
    sim <- recovery(sim)
  }
  return(sim)
}

# Initialise test sim
```

```r
sim1 <- initialiseSim(S0 = 1, I0 = 1, R0 = 0)

# Infection event, expect 1 fewer S and 1 more I.
sim2 <- nextEvent(sim1, TRUE)
expect_equal(sim2$S, 0)
expect_equal(sim2$I, 2)

# Recovery event, expect 1 fewer I and 1 more R.
sim3 <- nextEvent(sim1, FALSE)

expect_equal(sim3$I, 0)
expect_equal(sim3$R, 1)
```

We now need to redefine `runSim()` to account for these modified functions.

Code S11: Redfine the runSim function.

```r
runSim <- function(sim, beta, gamma, tf){
  # Run the simulation until time reaches
  #   tf.
  while (sim$time < tf) {

      sim <- addNew(sim)

      rates <- calcRates(sim, beta, gamma)

      # Increment the current time.
      sim$time <- sim$time + rates$dt

      # If we have reached time tf, finish the simulation
      if (sim$time > tf) {
          break
      }

      # Do the next event.
      event <- chooseEvent(rates$pf1, rates$pf)
      sim <- nextEvent(sim, event)

      # If the epidemic is extinct, end the simulation
      if (sim$I == 0) {
          break
      }
  }
  results <- data.frame(time = sim$ta, S = sim$Sa, I = sim$Ia, R = sim$Ra)
  return(results)
}
```

Pick a smart parameter for a deterministic result

In the same way that we used simple parameters values in Code S4, we can often find simple cases for which our stochastic functions become deterministic. For example, samples from $X \sim \text{Bernoulli}(p)$ will always be zeroes for $p = 0$ or ones for $p = 1$. In the case of the function `chooseEvent()`, this function is deterministic if `pf1` (the infection rate) is zero in which case the function will return `FALSE` i.e. if the infection rate is zero, the next event will not be an infection event. The function is also deterministic if `pf1` is equal to `pf`. This occurs when `pf2`, the recovery rate, is zero and therefore the next rate will certainly be an infection rate.

Code S12: A stochastic function can output deterministically if you can find the right parameter set.

```
expect_equal(chooseEvent(0, 1), FALSE)
expect_equal(chooseEvent(0, 5), FALSE)
expect_equal(chooseEvent(1, 1), TRUE)
expect_equal(chooseEvent(5, 5), TRUE)
```

Test all possible answers (if few)

Working again with a simple parameter set, there are some cases where the code is stochastic, but with a small, finite set of outputs. So we can run the function exhaustively and check it only returns possible outputs. For the function `chooseEvent()` this is trivially true; for any valid values of `pf1` and `pf` it should return either `TRUE` or `FALSE`.

Code S13: Testing stochastic output when it only covers a few finite values.

```
out1 <- replicate(300, chooseEvent(1, 1))
out2 <- replicate(300, chooseEvent(0, 1))
out3 <- replicate(300, chooseEvent(1, 2))

expect_true(all(out1 %in% c(TRUE, FALSE)))
expect_true(all(out2 %in% c(TRUE, FALSE)))
expect_true(all(out3 %in% c(TRUE, FALSE)))
```

Use very large samples for the stochastic part

While the previous example worked well for a small set of possible outputs, testing can conversely be made easier by using very large numbers. This typically involves large sample sizes or numbers of stochastic runs. For example, if `pf1` is positive but smaller than `pf`, we expect the `chooseEvent()` to sometimes return `TRUE` and sometimes to return `FALSE`. If we run the function only twice, we might get two `TRUE`s or two `FALSE`s. However, if we run the function thousands of times, and `pf1` is approximately half of `pf` (i.e. infection and recovery events are equally likely) then it is almost certain that we will get at least one of each logical value.

Code S14: Testing that the code does allow one individual to infect multiple individuals.

```
set.seed(10261985)
out3 <- replicate(5000, chooseEvent(1, 2))

# Check that both TRUE and FALSE are returned
#   and simultaneously check that ONLY TRUE or FALSE
#   is returned.
expect_true(setequal(out3, c(TRUE, FALSE)))
```

If we have an event that we know should never happen, we can use a large number of simulations to provide stronger evidence that it does not stochastically occur. However, it can be difficult to determine how many times is reasonable to run a simulation, especially if each run of the simulation is time consuming. This strategy works best when we have a specific bug that occurs relatively frequently (perhaps once every ten simulations or so). If the bug occurs every ten simulations and we have not fixed it, we would be confident that it will occur at least once if we run the simulation 500 or 1000 times. Conversely, if the bug does not occur even once in 500 or 1000 simulations then we can be fairly sure we have fixed it. As an example of this, the number of susceptibles in an SIR model should never increase. So we can run the simulation a number of times and check that it does not occur. However, without a previously observed bug, it is impossible to know how many runs of the simulation we would need to run before finding the bug, so in this example we will

simply run 100.

Code S15: Assessing if a bug fix was a likely success with large code runs, when the bug was appearing relatively frequently.

```
set.seed(11121955)
sim <- initialiseSim(100, 4, 0)

out <- replicate(100, runSim(sim, 0.1, 0.04, 30), simplify = FALSE)

# Does S ever increase?
#   This returns TRUE if every element of the S vector is
#   the same size or smaller than the previous element.
decreasing <- sapply(out, function(x) all(diff(x$S) <= 0))
expect_true(all(decreasing))
```

5 Further testing

Test incorrect inputs

As well as testing that functions work when given the correct inputs, we must also test that they behave sensibly when given wrong ones. This typically involves the user inputting argument values that do not make sense. This may be, for example, because the inputted argument values are the wrong class, in the wrong numeric range or have missing data values. Therefore it is useful to test that functions fail gracefully if they are given incorrect inputs. This is especially true for external, exported functions, available to a user on a package's front-end. However, it is not always obvious what constitutes an 'incorrect value' even to the person who wrote the code. In some cases, inputting incorrect argument values may cause the function to fail quickly. In other cases code may run silently giving false results or take a long time to give an error. Both of these cases can be serious or annoying and difficult to debug afterwards.

Often for these cases, the expected behaviour of the function should be to give an error. There is no correct output for an epidemiological model with a negative number of susceptible individuals. Instead the function should give an informative error message. Often the simplest solution is to use defensive programming and include argument checks at the beginning of functions. We then have to write slightly unintuitive tests for an expression where the expected behaviour is an error! If the expression does not throw an error, the test should throw an error (as this is not the expected behaviour). Conversely, if the expression does throw an error, the test should pass and not throw an error. We can use the `expect_error()` function for this task. This function takes an expression as its first argument and reports an error if the given expression does not throw an error as expected.

We can first check that the `calcRates()` function sensibly handles the user inputting a string instead of a number for the `beta` parameter. Because this expression throws an error, `expect_error()` does not throw an error and the test passes.

Code S16: Testing incorrect parameter inputs.

```
sim <- initialiseSim(100, 4, 0)
expect_error(calcRates(sim, beta = 'beta', 0.04))
```

Now we contrast what happens if the user inputs a negative number for the β parameter.

Code S17: A failing test for incorrect pathogen inputs

```
> sim <- initialiseSim(100, 4, 0)
> expect_error(calcRates(sim, beta = -1, 0.04))
NAs producedError: `calcRates(sim, beta = -1, 0.04)` did not throw an error.
```

The call to `calcRates()` did not throw an error and therefore `expect_error()` did throw an error. The easiest way to protect against this would be to use defensive programming and check the arguments. We demonstrate this in Code S18. Here we have added two checks that confirm that `beta` and `gamma` are positive. Given that `calcRates()` is run thousands of times (once per iteration) it might be more sensible to run these checks only once at the top of `runSim()`, but we are showing them here to avoid pasting the longer function again and for consistency with the previous two examples.

Code S18: Add defensive programming so that calcRates fails when it should do.

```
calcRates <- function(sim, beta, gamma){
  stopifnot(beta >= 0)
  stopifnot(gamma >= 0)
  rates <- list()
  # Infection rate.
  rates$pf1 <- beta * sim$S * sim$I
  # Transmission rate
  rates$pf2 <- gamma * sim$I
  # Total rate of all events
  rates$pf <- rates$pf1 + rates$pf2
  # Draw waiting time for this event.
  rates$dt <- rexp(1, rate = rates$pf)
  return(rates)
}

sim <- initialiseSim(100, 4, 0)
expect_error(calcRates(sim, beta = -1, 0.04))
```

Test special cases

When writing tests it is easy to focus on standard behaviour. However, bugs often occur at *special cases* — when the code behaves qualitatively differently at a certain parameter value of a parameter or for certain combinations. For example, in *R*, selecting two or more columns from a matrix e.g. `my_matrix[,2:3]` returns a matrix while selecting one column, e.g. `my_matrix[,2]` returns a vector. Code that relies on the returned object being a matrix would fail in this special case. These special cases can often be triggered with parameter sets that are at the edge of parameter space. This is where understanding of the functional form of the model can help. Consider a function `divide(x, y)` that divides `x` by `y`. We could test this function by noting that `y * divide(x, y)` should return `x`. If we write code that tests standard values of `x` and `y` such as `(2 * divide(3, 2)) == 3` we would believe the function works for nearly all values of division, unless we ever try `y = 0`.

In our example we might want to test whether the code works with $\beta = 0$ and $\gamma = 0$ (Code S19). We again run the simulation and test that the macro properties of the output are correct i.e. that the function returns a data frame with four columns and no NAs. While setting `beta = 0` works fine, setting `gamma = 0` does not.

Code S19: Demonstrate a failing test.

```
> sim <- initialiseSim(100, 4, 0)
> simulation1 <- runSim(sim, beta = 0, gamma = 1, 10)

> # Check the shape of the output
```

```
> expect_true(inherits(simulation1, 'data.frame'))
> expect_equal(ncol(simulation1), 4)

> # Test for NAs
> expect_true(all(!is.na(simulation1)))

> simulation2 <- runSim(sim, beta = 1, gamma = 0, 10)
Error in while (sim$time < tf) { : missing value where TRUE/FALSE needed
```

In general, requesting a simulation with a recovery rate of zero is not without meaning; this is not an objectively wrong use of the function. The error occurs because once all the individuals are in the infectious class, both the infection rate is zero ($\beta SI = 1 \times 0 \times 100 = 0$) and the transmission rate is zero ($\gamma I = 0 \times 100 = 0$). Therefore in the `rexp(1, rate = rates$pf)` in `calcRates`, we are trying to draw a random number from an exponential distribution with a rate of zero, which is undefined. Therefore `rates$dt` is set as `NaN`, `sim$time` subsequently becomes `NaN` and the expression `while (sim$time < tf)` errors because `NaN < 10` returns an `NA` rather than either a `TRUE` or a `FALSE`. For special cases like this, it may be rather subjective what the correct behaviour should be. It might be appropriate to simply declare that this parameter value is not allowed; setting `gamma` as `0` means this is no longer an SIR model but is instead an SI model. This should be stated in the documentation and the function should throw an error. This is what we will do here (Code S20). We change our defensive lines in `calcRates()` so that `gamma` must be greater than 0 rather than greater or equal than zero. We then add a test that checks that this argument is checked properly.

Code S20: Use defensive programming to check for gamma = 0.

```
calcRates <- function(sim, beta, gamma){
  stopifnot(beta >= 0)
  stopifnot(gamma > 0)
  rates <- list()
  # Infection rate.
  rates$pf1 <- beta * sim$S * sim$I
  # Transmission rate
  rates$pf2 <- gamma * sim$I
  # Total rate of all events
  rates$pf <- rates$pf1 + rates$pf2
  # Draw waiting time for this event.
  rates$dt <- rexp(1, rate = rates$pf)
  return(rates)
}

sim <- initialiseSim(100, 4, 0)


expect_error(runSim(sim, beta = 1, gamma = 0, 10),
             'gamma > 0 is not TRUE')
```

Finally we might also want to check that the function works with very large values of `beta` and `gamma`.

Code S21: Check very large values of beta and gamma.

```
sim <- initialiseSim(100, 4, 0)
simulation1 <- runSim(sim, beta = 1e5, gamma = 1, 10)

# Check the shape of the output
expect_true(inherits(simulation1, 'data.frame'))
expect_equal(ncol(simulation1), 4)
```

```r
# Test for NAs
expect_true(all(!is.na(simulation1)))


simulation2 <- runSim(sim, beta = 1, gamma = 1e5, 10)

# Check the shape of the output
expect_true(inherits(simulation2, 'data.frame'))
expect_equal(ncol(simulation2), 4)

# Test for NAs
expect_true(all(!is.na(simulation2)))


simulation3 <- runSim(sim, beta = 1e5, gamma = 1e5, 10)

# Check the shape of the output
expect_true(inherits(simulation3, 'data.frame'))
expect_equal(ncol(simulation3), 4)

# Test for NAs
expect_true(all(!is.na(simulation3)))
```

5.1 Session Info

```r
sessionInfo()
```

```
## R version 4.0.2 (2020-06-22)
## Platform: x86_64-pc-linux-gnu (64-bit)
## Running under: Ubuntu 20.04.1 LTS
##
## Matrix products: default
## BLAS:   /usr/lib/x86_64-linux-gnu/blas/libblas.so.3.9.0
## LAPACK: /usr/lib/x86_64-linux-gnu/lapack/liblapack.so.3.9.0
##
## locale:
##  [1] LC_CTYPE=en_GB.UTF-8       LC_NUMERIC=C               LC_TIME=en_GB.UTF-8
##  [4] LC_COLLATE=en_GB.UTF-8     LC_MONETARY=en_GB.UTF-8    LC_MESSAGES=en_GB.UTF-8
##  [7] LC_PAPER=en_GB.UTF-8       LC_NAME=C                  LC_ADDRESS=C
## [10] LC_TELEPHONE=C             LC_MEASUREMENT=en_GB.UTF-8 LC_IDENTIFICATION=C
##
## attached base packages:
## [1] stats     graphics  grDevices utils     datasets  methods   base
##
## other attached packages:
## [1] reshape2_1.4.4 testthat_2.3.2 ggplot2_3.3.2  knitr_1.29     rmarkdown_2.3
##
## loaded via a namespace (and not attached):
##  [1] tinytex_0.24      tidyselect_1.1.0  xfun_0.17         remotes_2.2.0
##  [5] purrr_0.3.4       listenv_0.8.0     colorspace_1.4-1  vctrs_0.3.4
##  [9] generics_0.0.2    usethis_1.6.3     htmltools_0.5.0   yaml_2.2.1
## [13] rlang_0.4.7       pkgbuild_1.1.0    pillar_1.4.6      glue_1.4.2
## [17] withr_2.2.0       sessioninfo_1.1.1 lifecycle_0.2.0   plyr_1.8.6
```

```
## [21] stringr_1.4.0     munsell_0.5.0     gtable_0.3.0      future_1.18.0
## [25] devtools_2.3.1    codetools_0.2-16  memoise_1.1.0     evaluate_0.14
## [29] labeling_0.3      callr_3.4.4       ps_1.3.4          parallel_4.0.2
## [33] fansi_0.4.1       Rcpp_1.0.5        backports_1.1.10  scales_1.1.1
## [37] desc_1.2.0        pkgload_1.1.0     farver_2.0.3      fs_1.4.2
## [41] digest_0.6.25     stringi_1.5.3     bookdown_0.20     processx_3.4.4
## [45] dplyr_1.0.2       rprojroot_1.3-2   grid_4.0.2        cli_2.0.2
## [49] tools_4.0.2       magrittr_1.5      tibble_3.0.3      crayon_1.3.4
## [53] tidyr_1.1.2       pkgconfig_2.0.3   ellipsis_0.3.1    prettyunits_1.1.1
## [57] assertthat_0.2.1  rstudioapi_0.11   R6_2.4.1          globals_0.12.5
## [61] compiler_4.0.2
```

References

Frost, Simon. 2020. "Stochastic SIR Model (Discrete State, Continuous Time) in R." http://epirecip.es/epicookbook/chapters/sir-stochastic-discretestate-continuoustime/r.
